# Fragile X mental retardation protein regulates skeletal muscle stem cell activity by regulating the stability of *Myf5* mRNA

**DOI:** 10.1186/s13395-017-0136-8

**Published:** 2017-09-07

**Authors:** Ryo Fujita, Victoria Zismanov, Jean-Marie Jacob, Solène Jamet, Krum Asiev, Colin Crist

**Affiliations:** 1 Lady Davis Institute for Medical Research, Jewish General Hospital, Montreal, Quebec H3T 1E2 Canada; 2 Department of Human Genetics, McGill University, Montreal, Quebec H3A 1B1 Canada; 3 Department of Radiation Oncology, Jewish General Hospital, Montreal, Quebec H3T 1E2 Canada; 4 Current address: Unité Stroma, Inflammation and Tissue Repair, Institut Pasteur, Paris, 75724 France

**Keywords:** Muscle stem cell, Satellite cell, Fragile X mental retardation protein, Myogenic regulatory factor *Myf5*

## Abstract

**Background:**

Regeneration of adult tissues relies on adult stem cells that are primed to enter a differentiation program, while typically remaining quiescent. In mouse skeletal muscle, these features are reconciled by multiple translational control mechanisms that ensure primed muscle stem cells (MuSCs) are not activated. In quiescent MuSCs, this concept is illustrated by reversible microRNA silencing of *Myf5* translation, mediated by microRNA-31 and fragile X mental retardation protein (FMRP).

**Methods:**

In this work, we take advantage of FMRP knockout (*Fmr1* ^*−/−*^) mice to support the role for FMRP in maintaining stem cell properties of the MuSC. We compare the activity of MuSCs in vivo after acute injury and engraftment, as well as ex vivo during culture. We use RNA immunoprecipitation and 3’UTR poly-adenine (poly(A)) length assays to assess the impact of FMRP on the stability of transcripts for myogenic regulatory factors.

**Results:**

We show that RNA-binding FMRP is required to maintain the MuSC pool. More specifically, FMRP is required for stem cell properties of muscle stem cells, which include MuSC capacity to prime the myogenic program, their self-renewal, and their capacity to efficiently regenerate muscle. We provide evidence that FMRP regulation of MuSC activity occurs in part by the capacity of FMRP to directly bind *Myf5* transcripts and impact rates of *Myf5* deadenylation.

**Conclusions:**

Our results provide further evidence supporting a role for post-transcriptional silencing platforms by RNA-binding proteins in maintaining stemness properties of adult stem cells. In addition, deregulated MuSC activity in the absence of *Fmr1* may have implications for fragile X syndrome, which is associated with muscle hypotonia during infancy.

## Background

Regeneration of adult tissues relies on the activity of adult stem cells that are primed to enter a tissue-specific differentiation program. In the skeletal muscle, these adult stem cell properties are illustrated by “satellite cells,” named for their position underneath the basal lamina of myofibers [1]. Satellite cells express members of the paired homeodomain family of transcription factors, *PAX7*, and in subset of muscle, *PAX3* [2, 3]. During development, PAX3/PAX7 are important regulators of myogenic progenitor survival and are required to activate the expression of myogenic determination genes *Myf5* and *MyoD*, with consequent rapid muscle differentiation [4].

Quiescent satellite cells are notable for being primed to activate the myogenic program while remaining quiescent. These features are reconciled by translational control mechanisms regulating gene expression by which microRNAs and RNA-binding proteins prevent the translation of messenger RNAs (mRNAs) for myogenic regulatory factors *Myf5* and *MyoD* [5, 6], as well as cell proliferation regulators such as Dek [7]. Furthermore, some mRNAs, such as those for *Myf5*, accumulate in RNA granules present in the quiescent satellite cell, which serve as sites of mRNA storage. These RNA granules form in response to the phosphorylation of eIF2α, which is a translational control mechanism regulating global protein synthesis that is required to maintain satellite cell quiescence [8].

Translational control of gene expression is an important mechanism by which a cell rapidly responds to its environment. These responses can be mediated by the activity of RNA-binding proteins such as fragile X mental retardation protein (FMRP), which regulates mRNA translation, localization, and stability [9]. The precise mechanisms by which FMRP influences translation of bound mRNAs remain unclear, but its phosphorylation at serine residue 499 is correlated with its presence in stalled polyribosomes [10]. FMRP is also associated with the RNA-induced silencing complex (RISC), suggesting that FMRP inhibits translation, at least in part, in cooperation with the microRNA pathway [11–13]. Emerging evidence indicates that FMRP has bifunctional activity to either permit or prevent the translation of transcripts to which it binds. In hippocampal neurons, phosphorylated FMRP (P-FMRP) binds to the RISC complex associated with miR-125 to silence *PSD-95* mRNA as it is transported to the termini of dendrites for localized translation. Translation of *PSD-95* mRNA at dendritic spines requires the dephosphorylation of FMRP, which causes the dissociation of *PSD-95* mRNA from miR-125/RISC silencing [14].

P-FMRP is also present in quiescent muscle stem cells, where we proposed that it facilitates the reversible inhibition of *Myf5* translation by microRNA-31. Upon satellite cell activation, FMRP is dephosphorylated. Blocking the FMRP phosphatase PP2A with okadaic acid prevents the translation of accumulating *Myf5* transcripts and delays the activation of the myogenic program [5].

In this study, we use *Fmr1* ^*−/−*^ mice to further support a role for FMRP in the stem cell properties of the satellite cell. We propose a mechanism by which FMRP RNA binding activity promotes the stability of myogenic regulatory factors such as *Myf5*. In the absence of FMRP, satellite cells have reduced capacity to prime the myogenic program, rapidly activate, self-renew, and regenerate muscle.

## Methods

### Mice

Care and handling of animals were in accordance with the federal Health of Animals Act, as practiced by McGill University and the Lady Davis Institute for Medical Research. For muscle regeneration, 6- to 8-week-old mice were anesthetized by isoflurane (CDMV) inhalation and 50 μl of 10 μM cardiotoxin (ctx) (Sigma) was injected into the *tibialis anterior* (TA) muscle. At 21 days after injury, the muscles were harvested for analysis by immunofluorescence. Cell engraftment assays were performed as previously described [8]. Immunocompromised 8-week-old *Foxn1* ^*nu/nu*^ female mice (Jackson Laboratories) were used. Donor cells were engrafted into the TA muscle, 24 h after the hindlimbs were exposed to 18 Gy irradiation.

### Cell and single-fiber isolation and culture

Satellite cells were isolated from the abdominal and diaphragm muscle, or from the ctx-injured TA muscle, of 5- to 8-week-old *Pax3* ^*GFP/+*^ and *Pax3* ^*GFP/+*^ *;Fmr1* ^*−/−*^ mice (Jackson Laboratories) [15] as previously described [16] using a FACSAriaIII cell sorter (BD Biosciences) or with magnetic beads (MACS Satellite Cell Isolation Kit, together with anti-Integrin a-7 MicroBeads, Miltenyl Biotec). Isolated cells were cultured in 39% DMEM, 39% F12, 20% fetal calf serum (Life Technologies), and 2% UltroserG (Pall Life Sciences). Single fibers were isolated by trituration of 0.2% collagenase D (Sigma)-treated *extensor digitorum longus* (EDL) muscle of adult mice [5].

### Immunodetection

Immunofluorescence labeling of cultured satellite cells, single EDL myofibers, and transverse sections of TA muscle was performed as described previously [5, 8]. For immunolabeling with antibodies against GFP, TAs were fixed for 2 h in 0.5% paraformaldehyde at 4 °C and equilibrated overnight in 20% sucrose at 4 °C. Tissues were mounted in Frozen Section Compound (VWR) and flash frozen in a liquid nitrogen cooled isopentane bath. For immunoblotting, cell lysates were prepared as described previously [5]. Densitometry of immunoblots was performed with ImageJ.

Primary antibodies were against PAX7 (DSHB, Pax7-c), MYF5 (Santa Cruz, sc-302), MYOD (SantaCruz, sc-304), LAMININ (Sigma, L9393), embryonic MHC (DSHB, F1.652), and β-ACTIN (Sigma, A5441). Alexa Fluor-488 and Alexa Fluor-594 conjugated secondary anti-mouse or anti-rabbit antibodies (Life Technologies) were used for immunofluorescence. Neuromuscular junctions were labeled with Alexa Fluor-488 bungarotoxin (Life Technologies). 5-Ethynyl-2′-deoxyuridine (EdU) (Life Technologies) was administered by a single intraperitoneal injection (40 mg/kg). After 24 h, transverse sections of frozen TA muscle were fixed with 4% paraformaldehyde for 15 min and washed twice with 3% bovine serum albumin in PBS and permeabilized with 0.5% Triton in PBS. Staining was performed with the Click-it EdU Alexa Fluor 594 kit (Life Technologies) [17]. Images were acquired with an AxioImager M1 fluorescence microscope (Zeiss). Horseradish peroxidase (HRP) conjugated goat anti-mouse or anti-rabbit secondary antibodies (Jackson Immunoresearch) were used with the ECL Prime Western Blotting Detection reagents (GE Healthcare) to image immunoblots with ImageQuant LAS 4000 (GE Healthcare).

### RNA immunoprecipitation

To immunoprecipitate FMRP mRNA protein complexes, 5 × 10^5^ C2C12 cells were seeded in 10 cm plates. Twenty-four hours later, cells were transfected with 5 μg pCAG-GFP [18] (gift from Connie Cepko, Addgene #11150) (control) or pFRT-TODestFLAGHAhFMRP [19] (FLAG-hFMRP) (gift from Thomas Tuschl, Addgene #48690) using jetPRIME transfection reagent (Polyplus tranfection) according to manufacturer’s instructions. Twenty-four hours after transfection, cells were lysed with polysome lysis buffer. Lysate preparation and immunoprecipitation was performed as described [20] using antibodies against FLAG M2 (Sigma, F1804) or GFP (DSHB, 8H11) with the exception that after washes, the total immunoprecipitated RNA was isolated using TRIzol reagent, as described below.

### RNA analysis

RNA was isolated from satellite cells or after immunoprecipitation of FMRP from C2C12 cells with TRIzol reagent (Life Technologies) and treated with DNase (Roche). RNA was reverse-transcribed with Superscript III reverse transcriptase (Life Technologies). Primers for quantitative PCR were *Pax7*, forward 5′-CTCAGTGAGTTCGATTAGCCG-3′, reverse 5′-AGACGGTTCCCTTTGTCGC-3′; *Myf5*, forward 5′-CTGTCTGGTCCCGAAAGAAC-3′, reverse 5′-AAGCAATCCAAGCTGGACAC-3′; *MyoD*, forward 5′-CCCCGGCGGCAGAATGGCTACG-3′, reverse 5′-GGTCTGGGTTCCCTGTTCTGTGT-3′, *Myog*, forward 5′-CAACCAGGAGGAGCGCGATCTCCG-3′, reverse 5′-AGGCGCTGT GGGAGTTGCATTCACT-3′, *Myh3*, forward 5′-GCATAGCTGCACCTTTCCTC-3′, reverse 5′-GGCCATGTCCTCAATCTTGT-3′, and *Actb* forward 5′-AAACATCCCCCAAAGTTCTAC-3′ reverse 5′-GAGGGACTTCCTGTAACCACT-3′.

Levels of mRNA were measured using SYBR Green on a 7500 Fast Real Time PCR System (Applied Biosystems). Poly-adenine (polyA) length assays were performed with the Poly(A) Tail-Length Assay Kit (Affymetrix) using gene-specific primers for *Myf5*, 5-CAGCTTTGACAGCATCTAC-3′ and 5′-TAGATAAGTCTGGAGCTGGA-3′; *Pax7*, 5′-GACTCCATCAAGCCAGGAG-3′; and 5′-TAGTAGGCTTGTCCCGTTTC-3′.

### Statistical analysis

Graphical analysis is presented as mean ± SEM. At least three independent replicates of each experiment were performed. Significance was calculated by unpaired Student’s *t* tests with two-tailed *p* values: *p < 0.05, **p < 0.01, ***p < 0.001.

## Results

### *Fmr1* is required for the homeostasis of primed satellite cells

**Fig. 1:**
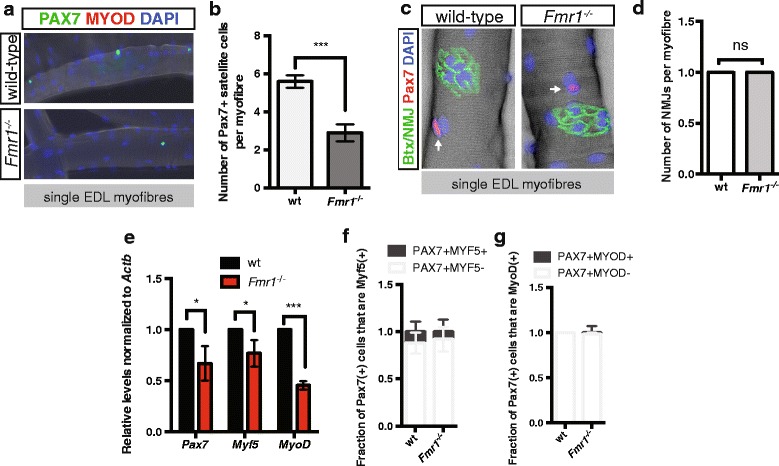
Skeletal muscle of *Fmr1* ^*−/−*^ mice have reduced numbers of satellite cells. **a** Immunostaining of PAX7 (green) on newly isolated single EDL myofibers isolated from adult wild-type (upper panel) and *Fmr1* ^*−/−*^ (lower panel) mice. **b** Numbers of PAX7-positive satellite cells per EDL myofiber isolated from wild-type (white) and *Fmr1* ^*−/−*^ (gray) mice. **c** Immunostaining of PAX7 (red) and bungarotoxin (Btx) labeling of neuromuscular junctions (NMJ) on single EDL myofibers isolated from wild-type and *Fmr1* ^*−/−*^ mice. **d** Numbers of NMJs per EDL myofiber. **e** Levels of *Pax7*, *Myf5*, and *MyoD* transcripts, relative to *Actb*, in satellite cells isolated from adult muscle of wild-type (wt, black) and *Fmr1* ^*−/−*^ (red) mice. **f** Fraction of PAX7(+) satellite cells that immunostain for MYF5 on single EDL myofibers isolated from muscle of wild-type (wt) and *Fmr1* ^*−/−*^ mice. **g** Fraction of PAX7(+) satellite cells that immunostain for MYOD on single EDL myofibers isolated from muscle of wild-type (wt) and *Fmr1* ^*−/−*^ mice. All values indicate mean (*n* ≥ 3) ± s.e.m. *p < 0.05, ***p < 0.001. ns not significant

To assess the impact of FMRP on the myogenic program, we began by examining satellite cells on single EDL myofibers isolated from wild-type and *Fmr1* ^*−/−*^ mice by immunostaining for PAX7. On average, three satellite cells are present for each EDL myofiber isolated from *Fmr1* ^*−/−*^ mice, compared to the normal complement of six satellite cells per EDL myofiber isolated from wild-type mice (Fig. 1a, b). While satellite cells are spread evenly throughout the myofiber, they are also often found in proximity to neuromuscular junctions (NMJs) [21, 22]. Furthermore, denervation leads to activation of satellite cells throughout the skeletal muscle [23]. In *Drosophila melanogaster*, knockout of *dFmr1* results in NMJs with altered structure and function [24]. We therefore asked whether the NMJ component of the satellite cell niche is altered in *Fmr1* ^*−/−*^ mice. Using bungarotoxin labeling, we show that single EDL myofibers from both wild-type and *Fmr1* ^*−/−*^ mice have a single NMJ, often in proximity to satellite cells. Abnormal NMJ morphologies were not observed (Fig. 1c, d).

Next, we asked whether intrinsic defects exist in satellite cells isolated from *Fmr1* ^*−/−*^ mice. We examined transcript levels for *Pax7*, *Myf5*, and *MyoD* in satellite cells isolated from the muscle of adult wild-type and *Fmr1* ^*−/−*^ mice. In the absence of FMRP, we show reduced levels of *Pax7*, *Myf5*, *and MyoD* (Fig. 1e). While *Myf5* and *MyoD* transcripts are present in quiescent satellite cells, translational control mechanisms ensure MYF5 and MYOD protein do not accumulate [5, 6, 8]. The absence of FMRP does not increase numbers of satellite cells expressing MYF5 or MYOD on single EDL myofibers (Fig. 1f, g).

### *Fmr1* is required for efficient satellite cell differentiation and self-renewal ex vivo

**Fig. 2:**
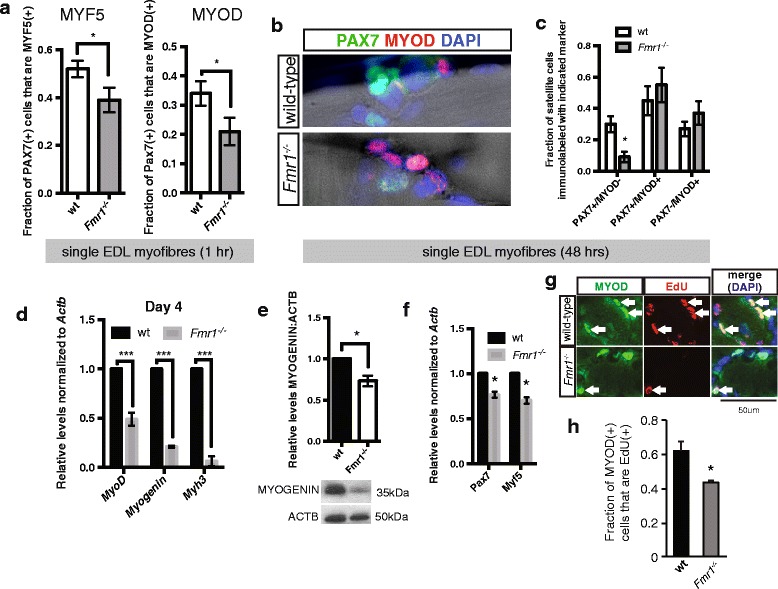
FMRP is required for efficient priming of muscle stem cells to rapidly activate the myogenic program, self-renewal, and differentiation. **a** Fraction of PAX7(+) satellite cells accumulating MYF5 (left) and MYOD (right) after 1 h culture of single EDL myofibers. **b** Representative immunostaining of PAX7 (green) and MYOD (red) on single EDL myofibers isolated from adult wild-type and *Fmr1* ^*−/−*^ mice after 48 h culture. **c** Numbers of satellite cells undergoing self-renewal (PAX7+MYOD−), activating the myogenic program (PAX7+MYOD+) and differentiating (PAX7−MYOD+) on single EDL myofibers isolated from adult wild-type (white) and *Fmr1* ^*−/−*^ (gray) mice after 2-day culture. **d** Levels of *MyoD*, *Myog*, and *Myh3* transcripts, relative to *Actb*, in satellite cells isolated from adult muscle of wild-type (wt, black) and *Fmr1* ^*−/−*^ (gray) mice, after 4-day culture. **e** Levels of MYOG protein, relative to ACTB, from cell lysates of 4-day cultured satellite cells isolated from muscle of wt (black) and *Fmr1* ^*−/−*^ (white) mice. Representative immunoblot is shown. MYOG levels are calculated by densitometry of three immunoblots. **f** Levels of *Pax7* and *Myf5* transcripts, relative to *Actb*, in satellite cells isolated from adult muscle of wild-type (wt, black) and *Fmr1* ^*−/−*^ (gray) mice, 2 days after ctx injury. **g** Representative images of EdU(+) MyoD(+) satellite cells present on transverse sections of TA muscle, 3 days after ctx injury. White arrows indicate MYOD(+) EdU(+) satellite cells. Scale bar, 50 μm. **h** Fraction of MYOD(+) satellite cells that are EdU(+), as indicated in **g**. All values indicate mean (*n* ≥ 3) ± s.e.m. *p < 0.05, ***p < 0.001

Since lower levels of *Myf5* and *MyoD* mRNAs are present in quiescent satellite cells isolated from the muscle of *Fmr1* ^*−/−*^ mice, we next asked if *Fmr1* ^*−/−*^ satellite cells are less primed to rapidly activate the myogenic program. Satellite cells on single EDL myofibers isolated from wild-type mice rapidly upregulate MYF5 and MYOD protein, within 1 h, but this rapid upregulation is delayed in satellite cells on EDL myofibers isolated from *Fmr1* ^*−/−*^ mice (Fig. 2a). To examine the role of FMRP during satellite cell entry into the myogenic program, EDL myofibers derived from *wt* and *Fmr1* ^*−/−*^ mice were cultured for 2 days and immunostained with antibodies against PAX7 and MYOD. Numbers of satellite cells undergoing self-renewal (PAX7+MYOD−) are reduced on EDL myofibers isolated from *Fmr1* ^*−/−*^ mice compared to those from wild-type mice, while those activating the myogenic program (PAX7+MYOD+) and undergoing differentiation (PAX7−MYOD+) are not significantly changed (Fig. 2b, c).

To examine later stages of differentiation, we cultured satellite cells isolated from the muscle of adult *Pax3* ^*GFP/+*^ and *Pax3* ^*GFP/+*^ *; Fmr1* ^*−/−*^ mice for 4 days. Differentiating satellite cells isolated from *Pax3* ^*GFP/+*^ *; Fmr1* ^*−/−*^ mice have reduced expression of myogenic regulatory factor mRNAs *MyoD and Myog*, as well as myosin heavy chain *Myh3* (Fig. 2d). The delay in differentiation of satellite cells isolated from *Pax3* ^*GFP/+*^ *; Fmr1* ^*−/−*^ mice is also evident by decreased levels of MYOG protein (Fig. 2e).

### *Fmr1* is required for satellite cell activity to regenerate muscle and self-renew in vivo

To ask whether defects observed in satellite cell self-renewal and differentiation are also observed in vivo, we next injured the TA muscle of wild-type and *Fmr1* ^*−/−*^ mice by intramuscular injection of cardiotoxin, a snake venom toxin that causes muscle fiber necrosis without affecting the viability of the satellite cell [25]. Two days after acute muscle injury by cardiotoxin injury in the TA muscle, satellite cells isolated from *Fmr1* ^*−/−*^ mice show reduced levels of *Pax7* and *Myf5* mRNAs, compared to those from wild-type mice (Fig. 2f). Satellite cells of *Fmr1* ^*−/−*^ mice also exhibit reduced frequency of proliferation, compared to wild-type mice, as shown by decreased incorporation of EdU (Fig. 2g, h).

**Fig. 3:**
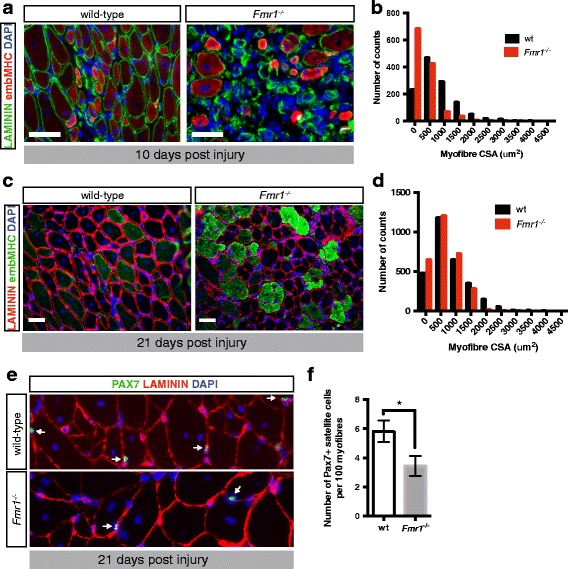
Delayed regeneration after acute injury of muscle in *Fmr1* ^*−/−*^ mice. **a** Immunostaining laminin (Lam, green), embryonic myosin heavy chain (embMHC, red) on transverse sections of TA muscle isolated from wild-type (left), and *Fmr1* ^*−/−*^ (right) mice 10 days after injury. Scale bar, white 50 μm. **b** Mean myofiber cross-section area (CSA) of wild-type (white) and *Fmr1* ^*−/−*^ *TA* muscle 10 days after injury. Numbers of myofibers with greater area than the indicated bin label are shown. **c** Immunostaining laminin (Lam, red), embryonic myosin heavy chain (embMHC, green) on transverse sections of TA muscle isolated from wild-type (left), and *Fmr1* ^*−/−*^ (right) mice 21 days after injury. Scale bar, white 50 μm. **d** Mean myofiber cross-section area (CSA) of wild-type (white) and *Fmr1* ^*−/−*^ TA muscle 21 days after injury. Numbers of myofibers with greater area than the indicated bin label are shown. **e** Immunostaining PAX7 (green) laminin (red) on transverse sections of *TA* muscle isolated from wild-type (upper panel) or *Fmr1* ^*−/−*^ (lower panel) 21 days after cardiotoxin injury. **f** Number of PAX7+ satellite cells present underneath the basal lamina, 21 days after injury of the *TA* muscle of wild-type (white) and *Fmr1* ^*−/−*^ (gray) mice. Values indicate mean (*n* ≥ 3) ± s.e.m. *p < 0.05

Ten days after muscle injury, we analyzed regeneration with antibodies against laminin and embryonic myosin heavy chain (embMHC). Evidence for a delay in muscle regeneration in the absence of *Fmr1* is indicated by increased expression of embMHC and smaller myofibers (Fig. 3a, b).

Twenty-one days after muscle injury, we analyzed regeneration with antibodies against PAX7, embMHC, and lamina. At this time point, wild-type mice downregulate embMHC in favor of adult isoforms. *Fmr1* ^*−/−*^ mice continue to exhibit smaller myofibers with perduring expression of embMHC (Fig. 3c, d). We also observe reduced numbers of satellite cells returning to their normal position underneath the basal lamina of the myofiber in regenerating muscle of *Fmr1* ^*−/−*^ mice, compared to that of wild-type mice (Fig. 3e, f).

**Fig. 4:**
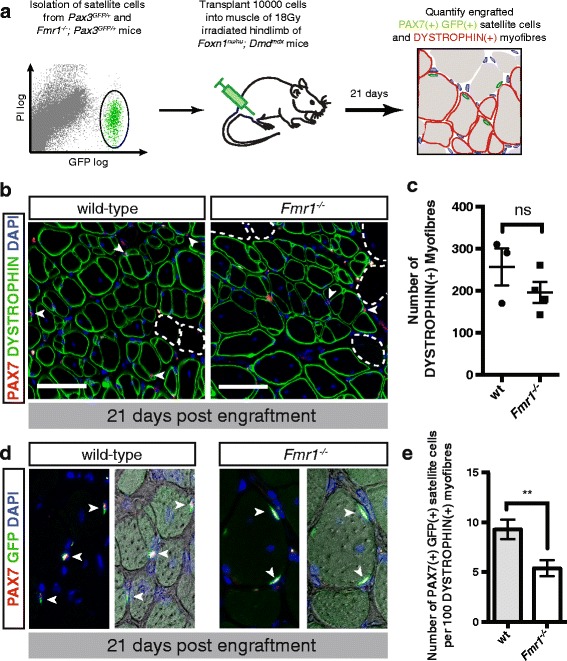
Satellite cells isolated from *Fmr1* ^*−/−*^ mice have reduced the capacity to self-renew and contribute to the satellite cell pool after intramuscular engraftment into a mouse model of Duchenne muscular dystrophy. **a** Schematic representation of cell engraftment. **b** Immunostaining dystrophin (green) and PAX7 (red) on transverse sections 21 days after engraftment of satellite cells that had been newly isolated from muscle of *Pax3* ^*GFP/+*^ (wild type; left) and *Pax3* ^*GFP/+*^ *; Fmr1* ^*−/−*^ (*Fmr1* ^*−/−*^; right) mice. Arrowheads indicate location of satellite cells. Dotted lines indicate position of dystrophin-negative myofibers. Scale bars (white) indicate 50 μm. **c** Numbers of dystrophin-positive myofibers 21 days after engraftment of 10,000 satellite cells isolated from *Pax3* ^*GFP/+*^ (wt) and *Pax3* ^*GFP/+*^ *; Fmr1* ^*−/−*^ (*Fmr1* ^*−/−*^) mice. **d** Immunostaining GFP (green) and PAX7 (red) on transverse sections 21 days after engraftment of satellite cells from muscle of *Pax3* ^*GFP/+*^ (wild-type; left) and *Pax3* ^*GFP/+*^ *; Fmr1* ^*−/−*^ (*Fmr1* ^*−/−*^; right) mice. Arrowheads indicate location of GFP(+) satellite cells of donor origin. **e** Numbers of PAX7-positive and GFP-positive satellite cells of donor origin, per 100 dystrophin-positive myofibers 21 days after engraftment of 10,000 satellite cells isolated from *Pax3* ^*GFP/+*^ (wt) and *Pax3* ^*GFP/+*^ *; Fmr1* ^*−/−*^ (*Fmr1* ^*−/−*^) mice. Values indicate mean (*n* ≥ 3) ± s.e.m. **p < 0.01. ns not significant

Although satellite cells in the muscle of *Fmr1* ^*−/−*^ mice have reduced numbers after acute injury, the initial satellite cell pool is also reduced (Fig. 1a, b). We therefore asked if FMRP is required for satellite cell self-renewal by following their fate after engraftment. Satellite cell engraftment into the *Dmd* ^*mdx*^ mouse model of Duchenne muscular dystrophy permits the assessment of the intrinsic ability of satellite cells to differentiate and restore dystrophin expression, as well as self-renew to populate the engrafted muscle with a donor-derived satellite cell pool. We isolated satellite cells from *Pax3* ^*GFP/+*^ and *Pax3* ^*GFP/+*^ *; Fmr1* ^*−/−*^ adult muscle and immediately engrafted 10,000 cells into the TA muscle of 18 Gy irradiated hindlimb of *Dmd* ^*mdx*^ *; Foxn1* ^*nu/nu*^ immunocompromised mice (Fig. 4a). Twenty-one days after engraftment, we show that satellite cells isolated from wild-type mice and *Fmr1* ^*−/−*^ mice have similar ability to contribute to hundreds of dystrophin-positive myofibers (Fig. 4b, c). In contrast, satellite cells isolated from *Pax3* ^*GFP/+*^ *; Fmr1* ^*−/−*^ mice have reduced capacity to repopulate the satellite cell pool, determined by immunofluorescence with antibodies against PAX7 and GFP (Fig. 4d, e).

### FMRP binds *Myf5* mRNA and regulates poly(A) tail length

**Fig. 5:**
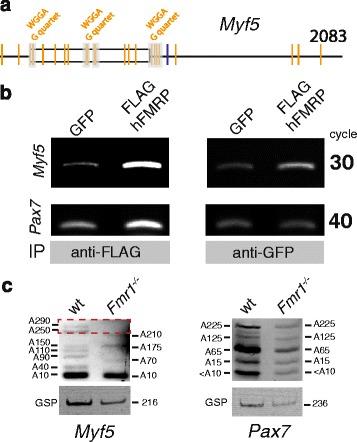
FMRP binds *Myf5* transcripts and affects rates of deadenylation. **a** Illustration of *Myf5* transcripts *(Mus musculus)* with FMRP binding WGGA (UGGA, AGGA) consensus sites indicated. Consensus sites which are within close proximity to each other and form potential FMRP binding G-quadraplex sites (gray) are indicated. One such G-quadraplex is in proximity to the microRNA-31 (miR-31) binding site on the 3’UTR of *Myf5*. **b** Immunoprecipitation (IP) of FMRP-mRNA complexes from C2C12 cells transfected with plasmids encoding GFP and FLAG-hFMRP. Immunoprecipitating antibodies are against FLAG-hFMRP (anti-FLAG, left) and GFP (anti-GFP, right). Amplification of *Myf5* (upper panels) and *Pax7* (lower panels) transcripts by semi-quantitative RT-PCR are shown, with thermocycle numbers indicated. **c** Poly(A) tail lengths of *Myf5* (left) and *Pax7* (right) transcripts amplified from total RNA isolated from 3-day cultures of satellite cells isolated from muscle of adult *Pax3* ^*GFP/+*^ (wild-type, wt) and *Fmr1* ^*−/−*^ mice. Estimated polyA lengths are indicated adjacent to each acrylamide gel. Gene-specific primers (GSP) against *Myf5* and *Pax7*, with product size indicated, are shown in the lower panels. A red dotted line indicates *Myf5* polyA lengths < 200 nucleotides present in wild-type but not in *Fmr1* ^*−/−*^ MuSCs

Together, our data suggests that FMRP regulates the efficiency of (a) priming quiescent satellite cells such that they rapidly activate the myogenic program, (b) self-renewal of satellite cells to maintain the satellite cell pool, and (c) differentiation of satellite cells to participate in regeneration of muscle. We therefore asked whether FMRP binds and regulates the stability of *Myf5* transcripts. The G-quadruplex structure is the best characterized FMRP binding sequence [26], and more recently, two independent FMRP CLIP sequencing datasets have permitted a more comprehensive examination of FMRP binding consensus sites [27]. *Myf5* mRNAs contain multiple FMRP consensus binding sites, which also assemble to form G-quadruplex structures (Fig. 5a). We therefore asked if FMRP directly binds *Myf5* mRNA. We transfected C2C12 cells with plasmids driving the expression of either GFP (control transfection) or FLAG epitope-tagged hFMRP [18] (FLAG-hFMRP) and subsequently performed RNA immunoprecipitation with antibodies against FLAG or GFP. *Myf5* transcripts were enriched when lysates were immunoprecipitated with antibodies against FLAG, compared with antibodies against GFP (Fig. 5b). *Pax7* transcripts are not enriched when lysates are immunoprecipitated with antibodies against FLAG or GFP (Fig. 5b).

If FMRP cooperates with RISC to reversibly repress *Myf5* translation in quiescent satellite cells and *PSD-95* in hippocampal neurons [5, 11, 12, 14], it remains unclear how excessive microRNA-dependent mRNA decay is avoided. Since RISC and RNA-binding protein-mediated silencing commonly occurs through the interaction with the CCR4/NOT1 deadenylase machinery [28, 29], we compared deadenylation of transcripts present in total RNA isolated from wild-type and *Fmr1* ^*−/−*^ satellite cells after 3-day culture. *Myf5* transcripts from satellite cells isolated from *Fmr1* ^*−/−*^ mice have shorter polyA tails than those isolated from wild-type mice. polyA tails of *Pax7* transcripts remain largely unchanged (Fig. 5c). Therefore, FMRP negatively regulates *Myf5* mRNA deadenylation in satellite cells.

## Discussion

Fragile X syndrome is the most commonly inherited form of intellectual disorder, affecting 1 in 1250 males and 2500 females [30]. Fragile X syndrome is a multi-organ disease that particularly leads to intellectual disorder, macroorchidism in males, and premature ovarian insufficiency in females. Fragile X syndrome is caused by the loss of function of *Fmr1* due to the increased expansion and hypermethylation of trinucleotide (CGG) repeats within the promoter of *Fmr1* [31]. FMRP is an RNA-binding protein and is widely expressed in human tissues, but particularly abundant in neurons. The absence of FMRP in neurons is believed to cause translation dysregulation defects in mRNA transport that is required for localized protein synthesis required for synaptic development and maturation.

Fragile X syndrome is also associated with muscle hypotonia (low muscle tone) during infancy [32]. The molecular mechanisms leading to muscle hypotonia are not clear. Given increasing evidence that satellite cells contribute to muscle homeostasis in newborn and young adult mice [33] and our previous observations supporting the cooperation of FMRP with microRNA-31 to regulate satellite cell quiescence and self-renewal [5], we asked whether *Fmr1* is required for satellite cell homeostasis. Using *Fmr1* ^*−/−*^ knockout mice, we demonstrate a role for FMRP in satellite cell activity. First, we show that skeletal muscle of *Fmr1* ^*−/−*^ knockout mice have approximately half the normal complement of satellite cells. Since numbers of satellite cells are not further reduced after an acute injury, it was difficult to determine whether self-renewal of satellite cells is normal in the absence of FMRP. We therefore used an engraftment assay to determine the fitness of donor muscle stem cells to differentiate and self-renew to repopulate the satellite cell pool. We show that satellite cells without FMRP retain their ability to differentiate, but have reduced capacity to self-renew and repopulate muscle with donor-derived satellite cells.

After an acute injury to the skeletal muscle, we also observe delays in regeneration of muscle. These delays may be in part due to a reduced number of satellite cells available to take part in skeletal muscle regeneration, but also in the intrinsic ability of *Fmr1* ^*−/−*^ satellite cells to activate the myogenic program and differentiate to repair muscle. We show that *Fmr1* ^*−/−*^ satellite cells are defective in two additional properties. First, quiescent *Fmr1* ^*−/−*^ satellite cells are less able to prime the myogenic program and show reduced levels of *Myf5* and *MyoD* transcripts, which are normally present but translationally silenced. Next, we provide evidence that *Fmr1* ^*−/−*^ satellite cells also take longer to activate the expression of *MYF5* and *MYOD* protein, which normally occurs within 1 h after isolation of single EDL myofibers. Second, *Fmr1* ^*−/−*^ satellite cells have reduced capacity to differentiate, illustrated by delayed expression of MYOD, MYOG and MYH3.

Altogether, we provide evidence that FMRP is required for satellite cells to (a) be primed for rapid activation, (b) efficiently execute the myogenic program to repair muscle after acute injury, and (c) self-renew. Here, we propose a model by which these three qualities of the satellite cell are influenced by the ability of FMRP to bind and regulate the stability and translation of *Myf5* transcripts. First, *Myf5* transcripts accumulate in the quiescent satellite cell, but remain translationally repressed [5]. How *Myf5* transcripts are repressed by the microRNA pathway, but remain stable, remains unclear. Our model by which FMRP directly binds and stabilizes *Myf5* transcripts is supported by a reduction of *Myf5* transcripts in *Fmr1* ^*−/−*^ quiescent satellite cells and immunoprecipitation of *Myf5* transcripts by FMRP in C2C12 myogenic cells. Second, during satellite cell activation, the continued stability of a pool of *Myf5* transcripts, facilitated by FMRP, would ensure rapid entry into the myogenic program and robust differentiation. Third, satellite cell self-renewal activity to repopulate the satellite cell pool after acute injury or after engraftment is also facilitated by FMRP, which is required to re-establish translational repression of *Myf5* transcripts to ensure MYF5 protein is not produced when satellite cells reacquire their quiescent state in their normal anatomical satellite position.

In our model, the bi-functional role of FMRP to repress *Myf5* translation during quiescence and permit *Myf5* translation during satellite cell activation could also be dependent on FMRP interaction with RISC. This switch is potentially mediated by FMRP phosphorylation, as we have previously proposed [5] in a similar way by which FMRP participates in reversible microRNA silencing observed at dendritic spines of hippocampal neurons [14]. FMRP interaction with other RNA-binding proteins and helicases [34] has also been proposed as mechanisms mediating the bi-functional role of FMRP.

It is unknown if FMRP influences rates of deadenylation of its target transcripts. Given a role for FMRP in reversible microRNA silencing of transcripts in neurons and muscle, we asked whether *Myf5* deadenylation rates in activated satellite cells is influenced by the presence or absence of FMRP. We show reduced accumulation of *Myf5* transcripts with long poly(A) tails in *Fmr1* ^*−/−*^ satellite cells, compared to wild-type satellite cells.

While we provide evidence that FMRP binds Myf5 and influences rates of *Myf5* deadenylation, it remains likely that FMRP also binds and regulates the stability of other myogenic regulatory factors. While we also observed lower expression levels of *MyoD*, *Myog*, and *Myh3* transcripts as *Fmr1* ^*−/−*^ satellite cells differentiate, it remains unclear whether FMRP directly binds these transcripts, or if their lower expression is due in part to reduced expression of upstream factors.

We also note that we observe defective satellite cell properties that lead to reduced differentiation and self-renewal, but we do not observe a collapse of regeneration or muscle homeostasis. This may be due in part to the redundancy observed between myogenic regulatory factors and the post-transcriptional silencing platforms that regulate their expression. We would expect, for example, that in genetic manipulations eliminating both *Fmr1* and tristetraprolin (*Ttp*), the protein product of which regulates the stability of *MyoD* to produce a more dramatic phenotype. In addition, while our ex vivo studies demonstrate intrinsic deficiencies in satellite cells, we cannot rule out the possibility that delays in regeneration we have observed are caused by defective cells that are normally required to support regeneration. Our results provide further evidence supporting a role for post-transcriptional silencing platforms by RNA-binding proteins in maintaining stemness properties of adult stem cells.

## Conclusions

In conclusion, we propose a model by which adult stem cells employ translational control of gene expression to prime activation of a differentiation program. In skeletal muscle, priming of the myogenic program is illustrated by accumulation of transcripts required for the myogenic program, including Myf5. In our model, the efficiency of translation of Myf5 is modulated by the activity of FMRP, which directly binds to Myf5 and regulates rates of poly-adenylation to ensure Myf5 stability. Genetic manipulations that eliminate Myf5 result in decreased capacity to prime the myogenic program, slower entry into the myogenic program, and delayed regeneration.

## Abbreviations

ctx: Cardiotoxin
EDL: Extensor digitorum longus
EdU: 5-Ethynyl-2′-deoxyuridine
embMHC: Embryonic myosin heavy chain
FMRP: Fragile X mental retardation protein
MuSCs: Muscle stem cells
NMJ: Neuromuscular junction
polyA: Poly-adenine
TA: Tibialis anterior
